# Sea ice and primary production proxies in surface sediments from a High Arctic Greenland fjord: Spatial distribution and implications for palaeoenvironmental studies

**DOI:** 10.1007/s13280-016-0894-2

**Published:** 2017-01-23

**Authors:** Sofia Ribeiro, Mikael K. Sejr, Audrey Limoges, Maija Heikkilä, Thorbjørn Joest Andersen, Petra Tallberg, Kaarina Weckström, Katrine Husum, Matthias Forwick, Tage Dalsgaard, Guillaume Massé, Marit-Solveig Seidenkrantz, Søren Rysgaard

**Affiliations:** 1 Department of Glaciology and Climate, Geological Survey of Denmark and Greenland, Øster Voldgade 10, 1350 Copenhagen K, Denmark; 2 Arctic Research Centre, Aarhus University, Ny Munkegade bldg. 1540, 8000 Aarhus C, Denmark; 3 Environmental Change Research Unit (ECRU), Department of Environmental Sciences, University of Helsinki, P.O. Box 65 (Viikinkaari 1), 00014 Helsinki, Finland; 4 IGN, Øster Voldgade 10, 1350 Copenhagen K, Denmark; 5 Department of Environmental Sciences, University of Helsinki, P.O. Box 65, 00014 Helsinki, Finland; 6 Fram Centre, Norwegian Polar Institute, P.O. Box 6606, Langnes, 9296 Tromsø, Norway; 7 Department of Geosciences, UiT – The Arctic University of Norway in Tromsø, Postboks 6050, Langnes, 9037 Tromsø, Norway; 8 Unité Mixte Internationale Takuvik, CNRS & Université Laval, Pavillon Alexandre-Vachon 1045, Avenue de la Médecine, Québec, QC G1V 0A6 Canada; 9 Department of Geoscience and Arctic Research Centre, Centre for Past Climate Studies, Aarhus University, Høegh-Guldbergs Gade, 28000 Aarhus C, Denmark; 10 Clayton H Riddell Faculty of Environment Earth and Resources, University of Manitoba, Winnipeg, MB R3T 2N2 Canada

**Keywords:** Arctic sea ice, Greenland fjords, HBIs, Primary production, Proxies, Silica

## Abstract

In order to establish a baseline for proxy-based reconstructions for the Young Sound–Tyrolerfjord system (Northeast Greenland), we analysed the spatial distribution of primary production and sea ice proxies in surface sediments from the fjord, against monitoring data from the Greenland Ecosystem Monitoring Programme. Clear spatial gradients in organic carbon and biogenic silica contents reflected marine influence, nutrient availability and river-induced turbidity, in good agreement with in situ measurements. The sea ice proxy IP_25_ was detected at all sites but at low concentrations, indicating that IP_25_ records from fjords need to be carefully considered and not directly compared to marine settings. The sea ice-associated biomarker HBI III revealed an open-water signature, with highest concentrations near the mid-July ice edge. This proxy evaluation is an important step towards reliable palaeoenvironmental reconstructions that will, ultimately, contribute to better predictions for this High Arctic ecosystem in a warming climate.

## Introduction

The thinning and retreat of Arctic sea ice is one of the most striking consequences of recent climate change, and has a very significant impact on Arctic ecosystem functioning (Wassmann et al. 2011). In the Arctic Ocean, sea ice retreat has led to an increase in light availability and a ca. 30% net primary production growth during the past two decades. However, this pattern is not uniform over the Arctic and a decline of about 15% in primary production has been reported for the Greenland Sea region (Arrigo and van Dijken 2015).

Studies along the latitudinal gradient in sea ice cover around Greenland show a significant impact of sea ice on productivity of both primary (Krause-Jensen et al. 2012) and secondary producers (Sejr et al. 2009). This suggests that any future changes in sea ice cover will impact the marine ecosystem. The polynyas and fjords of Northeast Greenland contribute significantly to the primary productivity of this region. Consequently, the need to understand ecosystem structure and function and to quantify possible changes for these marine systems influenced by a seasonal ice cover has led to monitoring efforts such as the Greenland Ecosystem Monitoring (GEM) Program. A large number of projects have focused on the Young Sound (e.g. Rysgaard and Glud 2007 and references therein). Today, this is one of the most studied High Arctic fjord systems in the world and can be considered a “natural laboratory”.

The Young Sound–Tyrolerfjord system is characterized by seasonal sea ice cover, with the ice-free season extending from approximately late July to late October (Sejr et al. 2011). The presence of sea ice determines the timing of pelagic primary production, which starts during the latest stage of sea ice melt and peaks when the ice disappears (Rysgaard et al. 1999). The surface water is eventually depleted in nutrients and the biomass of phytoplankton is restricted to the lower part of the photic zone where nutrients are still available. The extended sea ice cover combined with poor nutrient conditions limits productivity compared to other Greenland fjords such as the Godthåbsfjord system and the Disko Bay (Sejr et al. 2014; Meire et al. 2016). In addition to the phytoplankton production in the water column, sea ice microalgae that inhabit the sea ice matrix may contribute significantly to primary production, especially during spring and early summer. However, the biomass of ice algae and their productivity in Young Sound appears to be relatively low (Rysgaard et al. 2001). Benthic microalgae are also found in abundance on the seafloor where they contribute significantly to the total production of the outer part of Young Sound, especially at shallow depths (Attard et al. 2016).

Current research and monitoring activities in this High Arctic fjord system provide an invaluable insight into changes occurring at weekly, seasonal, and interannual time-scales, but in order to place recent changes in a longer-term context and to better constrain future scenarios for this region, it is necessary to reconstruct key parameters such as sea ice and primary production variability over multi-decadal to millennial time-scales. However, historical observation data for Northeast Greenland are particularly incomplete, and high-resolution marine sediment core records from this region are scarce. In order to address this, marine sediment-coring campaigns targeting the Young Sound, other Northeast Greenland fjords and the East Greenland shelf took place between 2013 and 2015, partly in connection with the Arctic Science Partnership (Greenlandic-Danish-Canadian Research collaboration^1^) and the TUNU Programme in Norway (Christiansen 2012).

Past environmental and climate variability prior to human observations can only be reconstructed from geological records by the use of indirect (proxy) data. Examples of primary production tracers are the organic carbon content of sediments (TOC), which can be used as a basic proxy for organic matter production and mineralization, as well as biogenic silica, an indicator of siliceous—mainly diatom—production. A relatively large number of proxies may be used to trace the presence and variability of sea ice (primarily dinoflagellate cysts, diatoms, and biomarkers, see de Vernal et al. 2013 and references therein). One such proxy is based on the abundance of a biomolecule termed IP_25_—ice proxy with 25 carbon atoms—a highly branched isoprenoid (HBI) lipid, which is synthesized in the sea ice matrix by some species of sea ice diatoms (Brown et al. 2014). Since the discovery of this Arctic sea ice proxy (Belt et al. 2007), an increasing number of palaeo-sea ice reconstructions based on IP_25_ have been produced (e.g. Massé et al. 2008; Müller et al. 2012; Belt et al. 2015), although reports of IP_25_ in Arctic fjords are rare (Cabedo-Sanz et al. 2013; Brown et al. 2015). It has been suggested that more detailed interpretations of the past sea ice conditions can be obtained through the comparison between IP_25_ and a related lipid biomarker (HBI III), which is likely produced by diatoms blooming in the open-water environment of the marginal ice zone (Belt et al. 2015; Smik et al. 2016). As such, the relative contributions to the sediment of endemic sea ice versus ice-edge phytoplankton can provide insights into past sea ice dynamics, and seasonal sea ice evolution.

The amount and quality of information available for the Young Sound fjord system provides an exceptional opportunity to evaluate the spatial distribution of these proxies against present-day conditions—a necessary first step for reliable palaeo-reconstructions. The main goal of this study is, thus, to provide a first evaluation of sedimentary signatures of primary production and seasonal sea ice along the fjord system (sediment core-top samples) in comparison with the present-day seasonal and interannual variability of these parameters (GEM data). This study defines a baseline for future palaeo-reconstruction studies using sediment core records from this region.

## Materials and methods

### Present-day conditions in the fjord

As part of the MarineBasis program, a hydrographic transect covering approximately 120 km is sampled every year in early August to quantify physical and biochemical properties in the Young Sound–Tyrolerfjord system (since 2003). Vertical profiles of salinity, temperature, oxygen, turbidity, and fluorescence are measured at approximately 30 stations using a Seabird 19+ CTD (Conductivity–Temperature–Depth) instrument with the appropriate sensors. In 2014, the monitoring program was complemented by additional measurements conducted in July, September, and October.

### Surface sediment samples

**Fig. 1:**
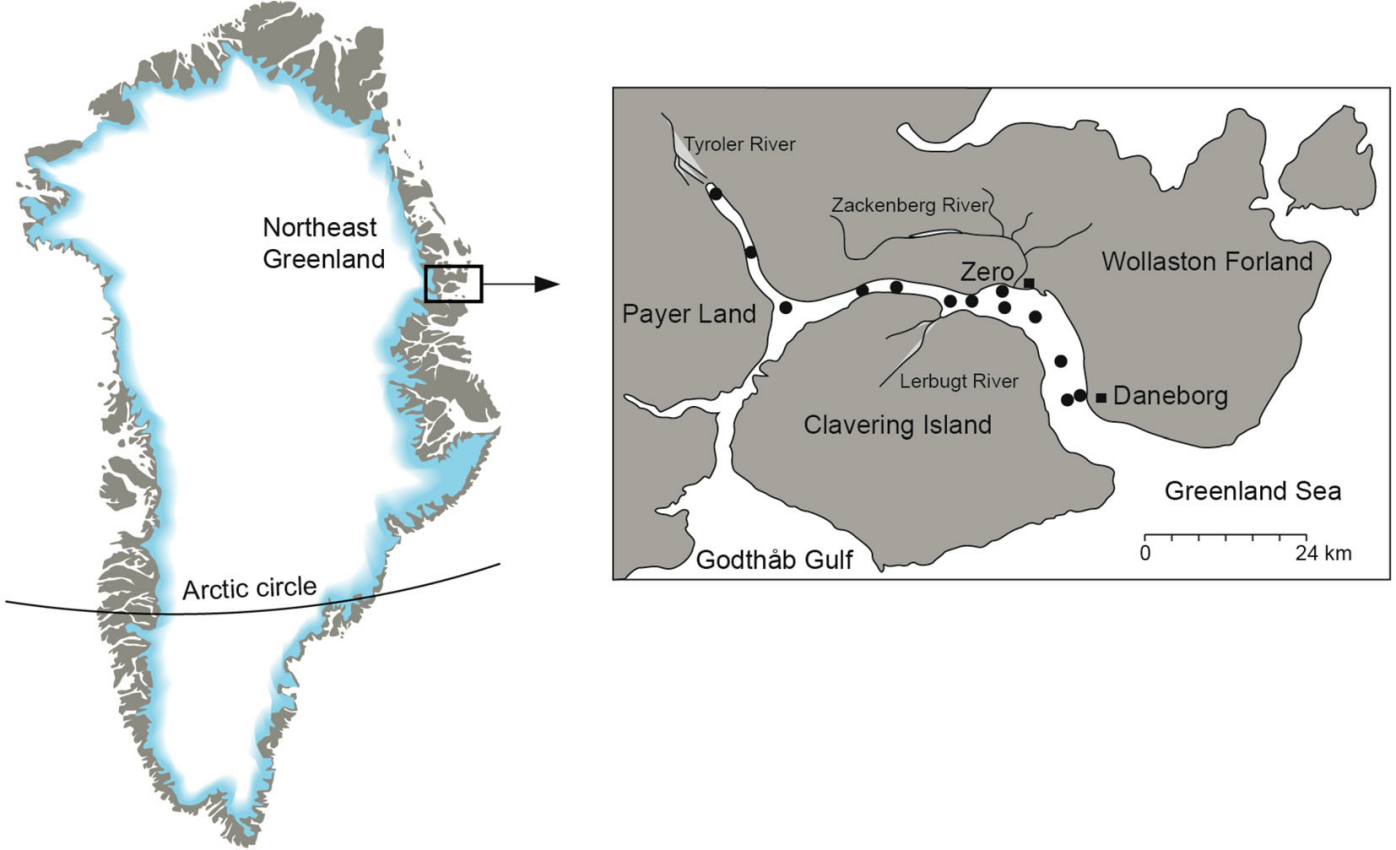
Location of Young Sound–Tyrolerfjord in Northeast Greenland. Surface sediment sample sites are marked as *dots*

A total of 13 sediment core-top samples were selected from four sediment-coring campaigns in the Young Sound in the years 2013–2015 (Table 1). Sample sites were chosen in order to cover the area from the Young Sound entrance to the inner part of Tyrolerfjorden, ranging from 60 to 344 m water depth (Fig. 1; Table 1). Three different coring devices were used (kayak corer, Rumohr lot and multi-corer) to recover undisturbed surface sediments. Samples represent the upper 0.5–1 cm of the cores and were freeze-dried and kept at −80 °C until further processing.

**Table 1:** Surface sediment samples selected for biogenic proxy analyses, listed from the outer Young Sound (YST1) to the innermost site in Tyrolerfjord (YST13)

Sediment sample	Core/site designation	Water depth (m)	Latitude	Longitude	Campaign	Coring device
YST1	SD60	60	74.3106	−20.2433	ARC/IGN15	Kayak corer
YST2	YS163	163	74.3097	−20.3	ARC/ASP13	Rumohrlot
YST3	HH13-25F	167	74.3667	−20.3412	UiT13	Multi-corer
YST4	YS3.14	150	74.4212	−20.5074	ARC/ASP14	Rumohrlot
YST5	HH13-23E	183	74.4341	−20.6483	UiT13	Multi-corer
YST6	Z60	60	74.4586	−20.6493	ARC/IGN15	Kayak corer
YST7	YS3.18	230	74.4343	−20.7573	ARC/IGN15	Kayak corer
YST8	HH13-21	240	74.4394	−20.8713	UiT13	Multi-corer
YST9	YSD	280	74.4644	−21.1864	ARC/IGN15	Kayak corer
YST10	HH13-19	344	74.4589	−21.3164	UiT13	Multi-corer
YST11	Tyro-8	160	74.4397	−21.6997	ARC/ASP14	Rumohrlot
YST12	Tyro-5	125	74.5025	−21.888	ARC/ASP13	Rumohrlot
YST13	Tyro100	100	74.6025	−22.086	ARC/IGN15	Kayak corer

*ARC* Arctic Research Center, *IGN* Department of Geosciences and Natural Resource Management at Copenhagen University, *ASP* Arctic Science Partnership, *UiT* The Arctic University of Norway, Department of Geosciences

### Grain-size distribution and age control

Primary particle size measurements were carried out by ultrasonically dispersing (2 min on a Bandelin UW 2200) the sediment in a sodium pyrophosphate-solution and analysing the dispersed particles on a Malvern Mastersizer E/2000. The dispersion was wet-sieved at 1 mm prior to analysis in the Malvern Mastersizer and no large particles were retained on the sieve. The grain-size distribution of the samples is given here as % clay, silt and sand.

In order to confirm that the sediments integrate a present-day signal of processes occurring in the fjord, all samples were analysed for the activity of ^210^Pb, ^226^Ra and ^137^Cs via gamma spectrometry at the Gamma Dating Center, Department of Geosciences and Natural Resource Management, University of Copenhagen. The measurements were carried out on Canberra ultralow-background Ge-detectors. ^210^Pb was measured via its gamma-peak at 46.5 keV, ^226^Ra via the granddaughter ^214^Pb (peaks at 295 and 352 keV) and ^137^Cs via its peak at 661 keV.

### Carbon analyses

Two sample sets, one for total carbon (TC) analysis and one for total organic carbon (TOC) analysis, were established to determine the carbon contents of the sediments. Freeze-dried sample materials were placed in 10-ml test tubes and the samples were then rinsed twice with distilled water to remove the salt residue. The samples in the TOC set were further treated with 10% hydrogen chloride (HCl) for 24 h to remove inorganic carbon. After 24 h, the samples were checked for acidity with litmus paper and as all of them maintained acidic solution, no further acid treatments were carried out. The samples were rinsed with deionized water until the pH of the supernatant reached that of the deionized water. Both the TC and the TOC set of samples were then freeze-dried again. A known quantity (9–150 mg) of freeze-dried material was weighed and packed into a tin (Sn) capsule. Carbon contents were analysed using a MICRO CUBE Elemental Vario elemental analyzer (EA) at the Laboratory of Geochemistry in the University of Helsinki with lake sediment (LKSD-4) as a reference material. Uncertainty, based on duplicate and triplicate measurements of the actual samples, is within ±0.1%.

### Biogenic silica analyses

The amorphous or biogenic Si (BSi) concentration in the sediment was analysed from freeze-dried sediment samples using the 1% Na_2_CO_3_ extraction method with mineral correction (DeMaster 1991). Samples of 30 ± 2 mg of sediment (2–3 replicates) were extracted in a 1% Na_2_CO_3_ solution in a water bath at 85 °C for 5 h. Subsamples of 1 ml were withdrawn after 180, 240 and 300 min and neutralized with 9 ml of 0.021 N HCl. The Si concentration in the subsamples was analysed using a spectrophotometer (Perkin Elmer lambda 25UV/VIS spectrometer) according to the blue ammonium molybdate method (Mullin and Riley 1955). The BSi concentration in each sediment sample was inferred from the intercept of the linear regression equation obtained by plotting the increase in Si against time, assuming that all amorphous, biogenic Si had dissolved after 2 h of extraction, while mineral Si dissolved continuously at a constant rate during the 5-h extraction (see e.g. Barão et al. 2015 for further details).

### HBI analyses (IP_25_ and HBI III)

Highly branched isoprenoid (HBI) analyses were conducted according to the procedure described in Belt et al. (2007). Prior to analytical treatment, an internal standard (7-hexylnonadecane) was added to ca. 0.5 g of freeze-dried and homogenized sediment. Total lipids were ultrasonically extracted (×3) using a mixture of dichloromethane (DCM:CH_2_Cl_2_) and methanol (MeOH) (2:1, v/v). Extracts were pooled together and the solvent was removed by evaporation under a slow stream of nitrogen. The total extract was subsequently re-suspended in hexane and purified through an open column chromatography (SiO_2_). Hydrocarbons (including IP_25_ and HBI III) were eluted using hexane. Procedural blanks and standard sediments were analysed in between every 15 samples. Hydrocarbon fractions were analysed using an Agilent 7890 gas chromatograph (GC) fitted with a 30-m fused silica Agilent HP5-MS column (0.25 mm i.d. and 0.25 µm phase thickness) and coupled to an Agilent 5975C Series mass selective detector (MSD). Oven temperatures were programmed as follows: 40–300 °C at 10°C min^−1^, followed by an isothermal interval at 300 °C for 10 min. The data were collected using Chemstation and analysed using the MassHunter quantification software. IP_25_ and HBI III were identified on the basis of retention time and comparison of mass spectra with authenticated standards. Abundances were obtained by comparison between individual GC–MS responses relative to those of the internal standard. Data are reported here in ng g^−1^.

## Results and discussion

### Present-day conditions in the fjord

**Fig. 2:**
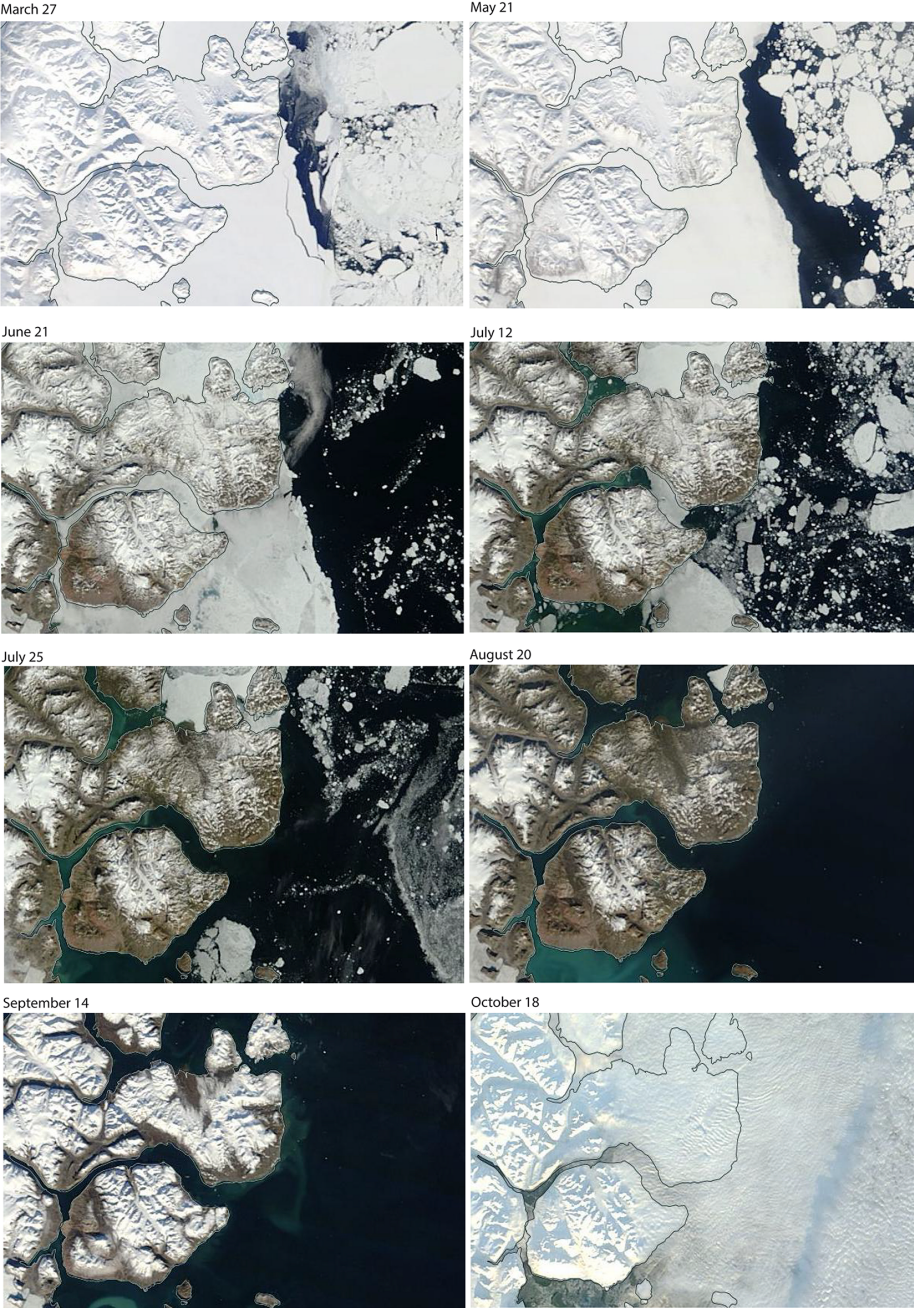
Seasonal sea ice conditions in the fjord (based on MODIS imagery) for the year 2014

Satellite images from 2014 show the typical seasonal sea ice development in Young Sound (Fig. 2). The area is covered by landfast sea ice that reaches a maximum around February and March. During May and June, the ice-free area outside Young Sound gradually expands. In June, meltwater ponds start to form on top of the sea ice, and in early July, open water is often visible in the inner part of the Tyrolerfjord. In late July, the remaining sea ice is flushed out of the fjord, which remains largely ice-free until October or November, when ice forms again depending on wind conditions (Fig. 2).

**Fig. 3:**
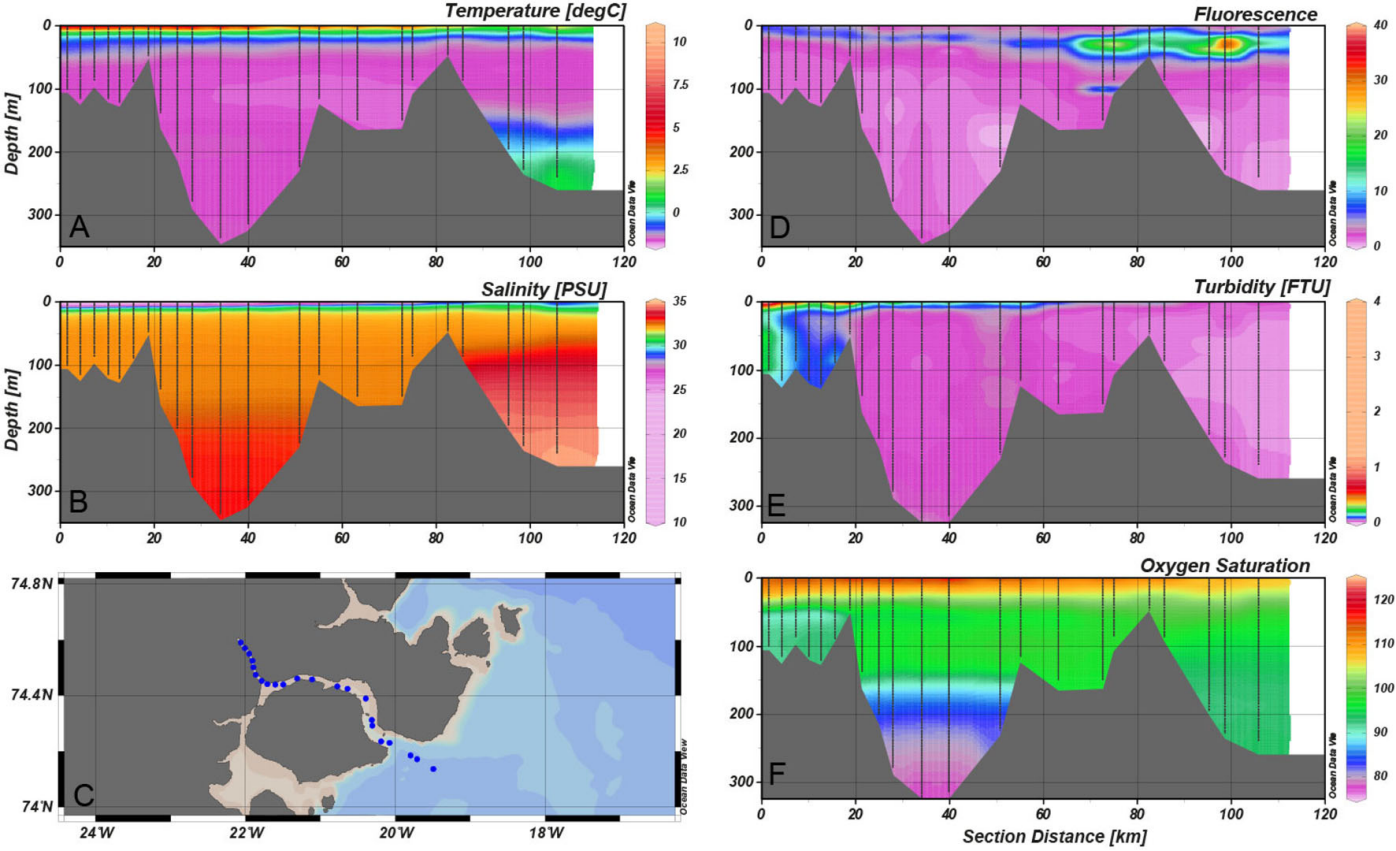
Contour plots of key hydrographical parameters along the fjord in early August (2014). CTD casts are marked as *black vertical lines* on the transects. **a** Temperature, **b** salinity, **c** *blue dots* show the location of the CTD stations, **d** fluorescence, **e** turbidity, **f** oxygen saturation

**Fig. 4:**
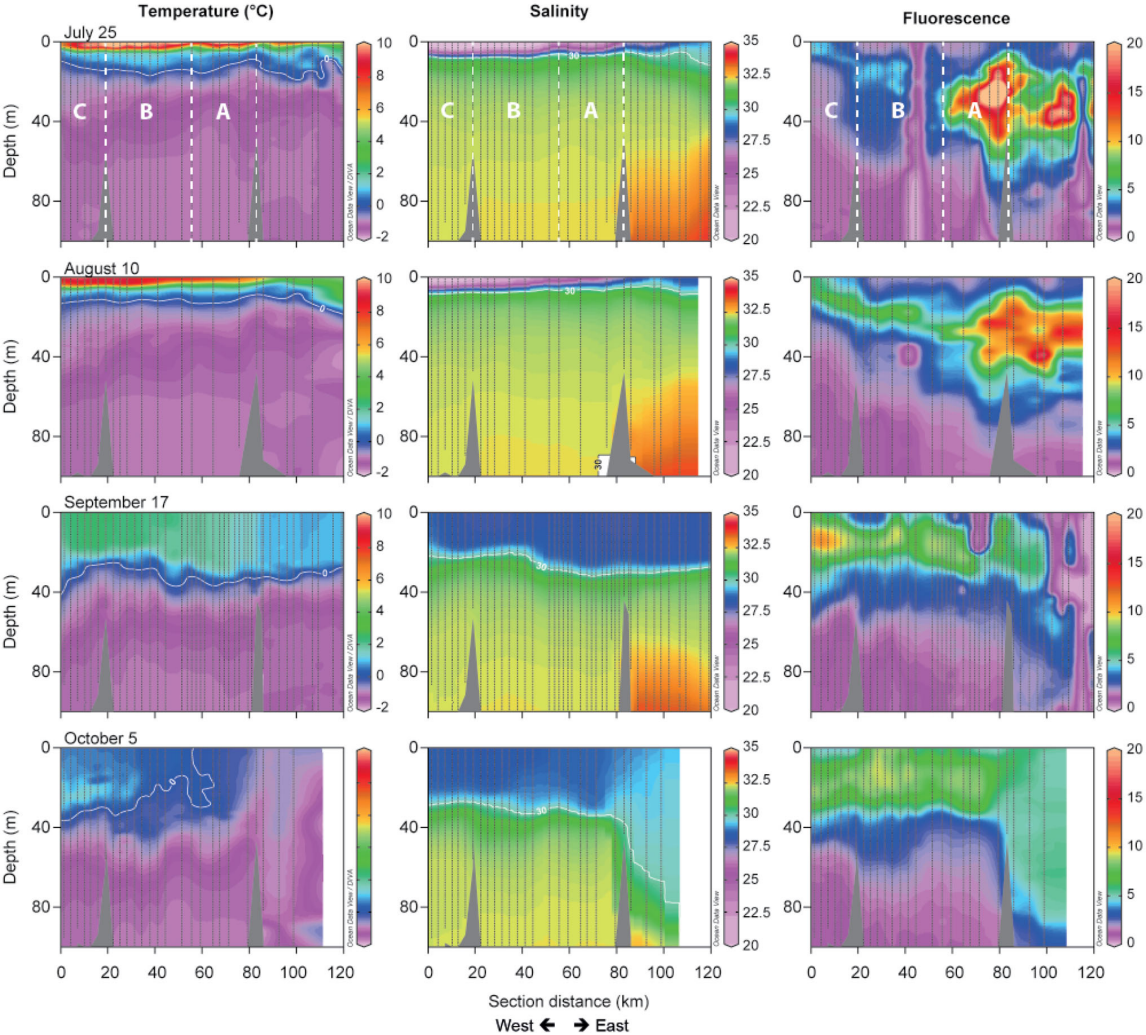
Evolution of key parameters (temperature, salinity and fluorescence) along the fjord transect (Fig. 3c) during the ice-free period (late-July–October 2014) down to 100 m water depth. CTD casts are marked as *black vertical lines*. The distribution of zones A–C is highlighted on the *top panels*

The surface salinity is influenced by the melting sea ice, which combined with meltwater from land creates a shallow 5–10 m lens of warm, low-salinity surface water (Fig. 3a, b). Lowest salinity is observed in the inner Tyrolerfjord where it may be below 15 psu and gradually increases towards the fjord entrance. As freshwater input from land typically terminates by early September, the surface layer becomes gradually mixed into deeper water and the freshwater is distributed more evenly in the upper 30–50 m of the water column (Figs. 3b, 4). In August, the three larger rivers (Tyroler River, Lerbugt River, and Zackenberg River; Fig. 1) all export large amounts of particles causing high turbidity, particularly in the inner part of the fjord (zone C) that is directly impacted by glacial rivers (Fig. 3e). The high turbidity absorbs most of the sunlight in the shallow surface lens, which is consequently heated to more than 10 °C. Under this warm surface layer, temperatures are below 0 °C, except on the shelf outside the fjord, where warm Atlantic subsurface water (Irminger Water) is found below 200 m depth (Fig. 3a). The high turbidity and consequently poor light conditions are reflected in very low levels of fluorescence—a proxy for phytoplankton biomass—in the inner fjord part. In August, the peak in fluorescence is typically found in the outer fjord due to a combination of more favourable light and nutrient conditions (Fig. 3d, zone A). Fluorescence levels decrease in the outer part of the fjord during the ice-free period (July–October) and increase slightly in the upper 40 m of the fjord waters (Fig. 4). Oxygen levels in the fjord are mostly close to saturation, with a slight oversaturation in areas of phytoplankton production (surface waters) and under-saturation equivalent to 70–80% in the bottom waters of the deepest basin (Fig. 3f, zone B).

### Surface sediment samples

#### Grain size and age control

**Fig. 5:**
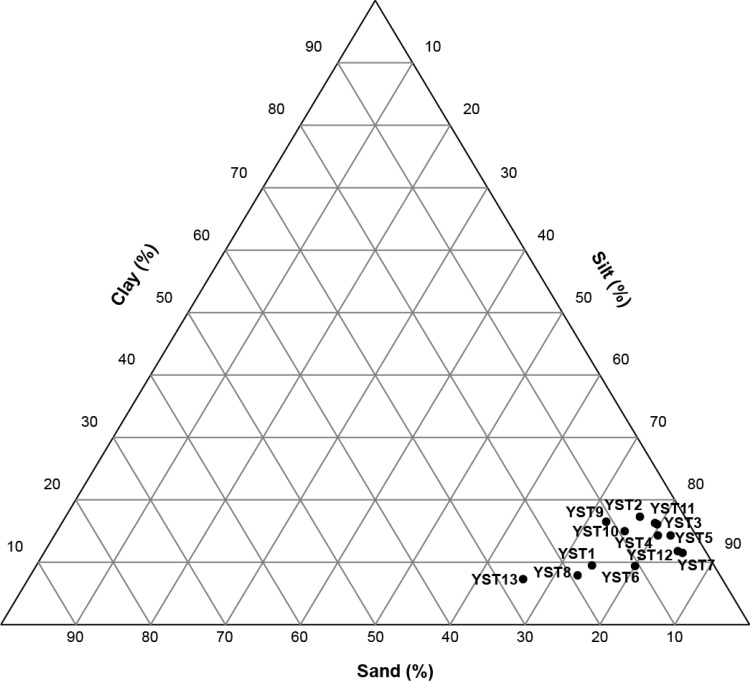
Grain-size distribution of the surface sediment samples

All surface sediment samples consisted of silt and sandy silt (Fig. 5), and the grain-size distribution was relatively uniform, not following a clear pattern with depth or distance in the fjord. Only the innermost fjord sample (YS13) contained more than 20% sand (Fig. 5).

The presence of unsupported ^210^Pb confirms that the sediment is recent, and has been deposited <100 years ago, most likely very recently. One sample (YST13) did not show measurable content of unsupported ^210^Pb but this may be caused by the sandy nature of the sample (Fig. 5). Additionally, ^137^Cs has been released into the environment since the 1950s and during the Chernobyl disaster of 1986, and the presence of this isotope in several of the samples confirms the recent nature of the material. However, the content of this isotope is generally low and close to the detection limit of about 2–3 Bq kg^−1^ and the absence in some of the samples therefore does not necessarily indicate deposition prior to the 1950s. On the contrary, preliminary dating of the sediment cores collected at sites YS4, YS7, YS9, and YS11, all show ^137^Cs activity further down-core, confirming that the upper 0–1 cm of sediment has been deposited within the last years to decade(s).

#### Organic carbon

**Fig. 6:**
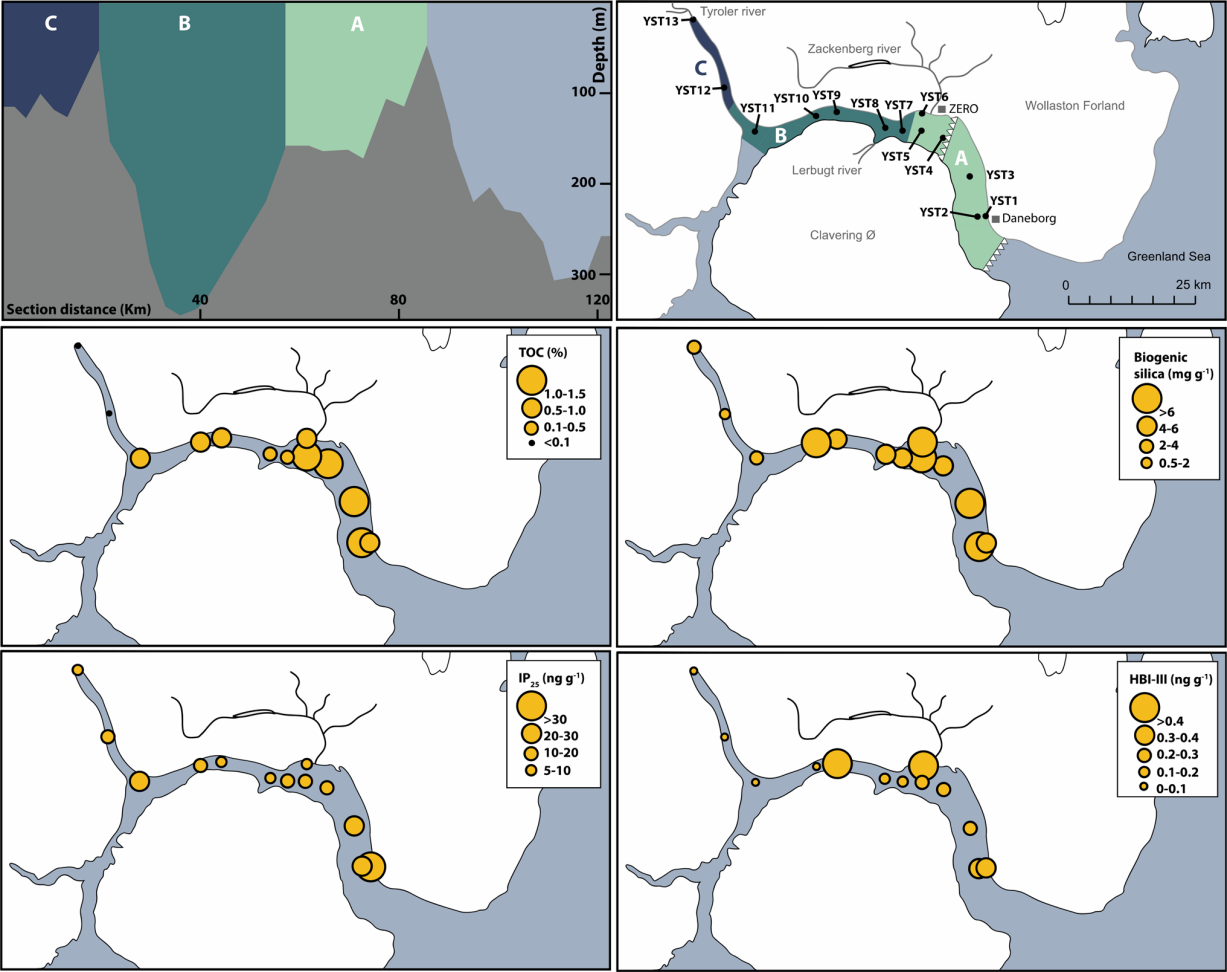
Spatial distribution and abundance of biogenic proxies in the fjord sediments. The distribution of zones A–C is given in the *top panels*. The *white series of triangles* show the approximate position of the July sea ice edge

Total organic carbon (TOC) results helped to define three zones within the fjord (Fig. 6). Zone A, consisting of the samples from the outermost part of the fjord (YST1 to YST6), had values between 0.7 and 1.5%. The samples from zone B, in the middle part of the fjord, had generally lower values than zone A (0.2–0.7%). Zone C consisted of the two innermost sites (YS12 and YS13) and had the lowest values of TOC < 0.1% (Fig. 6). The observed pattern in TOC is likely a result of several processes: seasonal sediment trap data from zone A show that more than 90% of the annual vertical flux of particulate organic matter occurs during the ice-free period, with nearly equal contributions from marine primary production and riverine input (Rysgaard and Sejr 2007). Since zone B and zone C are more influenced by the rivers, the contribution of terrestrial carbon is likely more important, but due to high sedimentation of inorganic particles, the organic carbon signal is “diluted” in the inner part of the fjord. Zone A of the fjord is the most productive, due to more favourable light and nutrient conditions (Murray et al. 2015). The main primary producers in the outer region of the fjord are phytoplankton (65%) and benthic algae (34%), while the contribution of sea ice algae is minimal (<1% according to Glud and Rysgaard 2007). Previous studies on carbon cycling in sediments from the outer Young Sound showed that about 70% of the organic carbon reaching the seafloor is oxidized (Thamdrup et al. 2007). However, at water depths higher than 80 m, carbon burial exceeds oxidation (Rysgaard and Sejr 2007).

The generally higher TOC values for YST1 to YST6 (zone A, Fig. 6) most likely reflect organic matter fluxes originating from the phytoplankton bloom that extends from outside the fjord and past the entrance sill (as seen by the fluorescence measurements, Figs. 3, 4), likely influenced by the vertical mixing across the shallow sill (Arendt et al. 2016). Interestingly, the two lowest values measured in this zone are for YST1 and YST6, which both lie at a depth of about 60 m, where carbon oxidation is expected to exceed burial (Thamdrup et al. 2007). The TOC values <0.1% for YST12 and YST13 are in good agreement with the high turbidity and low fluorescence measured in this area during the summer (Figs. 3, 6), which inhibits production.

#### Biogenic silica

The BSi concentration in the Young Sound–Tyrolerfjorden sediments varied between 1.3 and 9 mg g^−1^ DM Si (median 5.8 mg g^−1^), corresponding to 0.3 and 1.9 wt% SiO_2_ (median 1.2 wt% SiO_2_). Biogenic silica (BSi) refers to the amorphous Si content in sediments. It is mainly used as a proxy for siliceous microfossil abundance, and it has been shown to be a useful proxy for diatom abundance and productivity in aquatic systems (Conley and Schelske 2001). Measurements of BSi from marine sediments in the Arctic region are quite scarce, but BSi concentrations in the surface Young Sound sediments are comparable to those (<2%) measured from the Greenland Sea (Schlüter and Sauter 2000), and appear to be low compared to the study by Heikkilä et al. (2014) in surface sediment from the Hudson Bay system (2–11%).

The BSi content in the surface sediment followed a pattern similar but not identical to the TOC values. While the latter reflects the overall trends in primary productivity in the fjord (zone A being the most productive, and zone C the least productive), biogenic silica reflects the interplay between the available dissolved silica from glacial meltwater and light availability. Three major glacial rivers discharge into the fjord and the total combined runoff from land has been estimated to 0.9–1.4 km^3^ year (Rysgaard et al. 2004; Bendsten et al. 2014), with a great impact on salinity and influx of both sediments and macronutrients. Among the macronutrients, dissolved silica is exported in large quantities from the weathering of silicate rock by glaciers. Silica is an important nutrient for diatom growth, and hence a controlling factor in primary productivity in the Young Sound–Tyrolerfjord system. Although runoff is highest in the inner parts of the fjord, biological uptake of dissolved silica does not match its availability (Rysgaard et al. 1999) in zone C, possibly due to the high turbidity here (Fig. 3). Our analyses of biogenic silica content in the surface sediment samples show that the highest concentrations were instead measured in sediments from site YST6, near the Zackenberg river mouth, where dissolved silica concentrations are high but turbidity is relatively low (see Fig. 1 in Meire et al. 2016).

During the summer, phytoplankton assemblages are largely dominated by diatoms in the outer part of the fjord, while dinoflagellates and ciliates increase in abundance towards station YST7 and colonial chrysophytes become the most abundant group around station YST12 (data from summer 2012, see Krawczyk et al. 2015 for details). These groups of organisms have very different contributions to the sediment. All diatoms and some chrysophytes will enrich the BSi silica content in the sediments, whereas the majority of dinoflagellates, ciliates and their cysts are composed of refractory organic material. These differences in phytoplankton composition appear to be reflected in the BSi content of the sediments, as the highest BSi concentrations were generally found in zone A, with the exception of station YS10 (Fig. 6). Although biogenic silica contents in the sediments are a tracer of both planktic and benthic diatom productivity, all the sites studied here are expected to have a very low contribution from benthic microalgae, because these are mainly found at depths <30 m (Glud et al. 2002).

### Spatial distribution of sea ice proxies

IP_25_ concentrations ranged from 8 to 42 ng g^−1^ dry sediment (Fig. 6; Table 2). The presence of IP_25_ at all sites primarily reflects the seasonal nature of the ice cover in the fjord. This molecule is produced by diatoms thriving in the sea ice bottom (at 1–3 cm from the ice–water interface, according to Brown et al. 2011), and released from the sea ice matrix during ice melt. The source of IP_25_ has been identified by Brown et al. (2014) as pan-Arctic diatom species belonging to the genera *Haslea* (*H. spicula*/*crucerigeroides* and *H. kjellmanii*) and *Pleurosigma* (*P. stuxbergii* var. *rhomboides*). Within the *MarineBasis* framework of the GEM Programme, phytoplankton identification counts have been performed since 2004, and the dominant diatom species at the water/sea ice interface have also been reported for the Young Sound (Glud et al. 2007). Microscopic examination of sea ice core samples from the outer fjord region revealed the presence of IP_25_ producers as minor contributors to sympagic (in-ice) production, supporting an in situ source of this biomarker (our unpublished results).

**Table 2:** Overview of sedimentological and biogenic proxy results

Sample	Grain size			Radiometric data				Proxy data			
	Clay (%)	Silt (%)	Sand (%)	^210^Pb_exc_ (Bq kg^−1^)	SD	^137^Cs (Bq kg^−1^)	SD	TOC (%)	BSi (mg g^−1^)	IP_25_ (ng g^−1^)	HBI III (ng g^−1^)
YST1	9.5	74.2	16.3	32	6	4	3	0.65	5.12	41.90	0.34
YST2	17.3	76.7	6.0	104	13	31	3	1.33	6.15	20.69	0.35
YST3	16.1	79.7	4.3	170	26	17	4	1.47	6.44	21.01	0.25
YST4	14.3	80.6	5.1	197	38	0	0	1.46	4.20	13.05	0.26
YST5	14.3	82.3	3.4	63	13	24	5	1.02	6.67	13.76	0.24
YST6	9.4	80.0	10.6	34	8	2	1	0.70	6.79	7.97	0.56
YST7	11.5	85.3	3.2	187	37	0	0	0.50	4.00	13.67	0.15
YST8	7.9	73.0	19.0	43	30	3	8	0.21	4.99	9.21	0.11
YST9	16.5	72.6	10.9	195	28	0	0	0.66	5.80	9.54	0.65
YST10	15.0	75.9	9.2	372	75	45	9	0.54	7.06	14.38	0.00
YST11	16.3	79.3	4.5	148	30	0	0	0.90	3.10	21.93	0.04
YST12	11.8	84.6	3.7	41	21	10	4	N/A	1.28	12.67	0.03
YST13	7.3	66.1	26.6	0	0	3	1	N/A	2.53	9.22	0.00

With the exception of site YST11, where values are comparably elevated, IP_25_ concentrations in the sediments are generally low along the fjord; highest concentrations are found in the outer fjord region, where conditions are closer to those of a marine setting (Figs. 3, 4). This suggests that the fjord conditions are somewhat unfavourable for IP_25_-producing taxa. While no studies so far have been dedicated to investigate the distribution of IP_25_ in Arctic fjord sediments, it has been suggested that significant river runoff could hamper the growth of sea ice algae (Xiao et al. 2013). In the Young Sound–Tyrolerfjord system, it has been shown that during spring the sea ice environment becomes structurally very dynamic due to the large freshwater input from breaking rivers and snow melt (Glud et al. 2007). Atmospheric forcing can lead to episodes of freshwater percolation through the sea ice matrix and the formation of an extensive lens of low-salinity water under the ice, with a strong impact on the sea ice environment and its biota (Glud et al. 2007). In the absence of comparable data from marine sites outside the fjord, it is difficult to clearly assess the degree to which salinity may influence IP_25_ production in the study area, but we do not exclude this as a possible scenario.

Triene (HBI III) values ranged from 0 to 0.7 ng g^−1^ dry sediment (Fig. 6; Table 2). While zone C is characterized by the near absence of HBI III, abundances were higher in sediments from the middle and outer parts of the fjord (zones B and A). In contrast to IP_25_, whose production is confined to (benthic) diatoms living in the sea ice, HBI III is likely derived from marine open-water planktic diatoms [only known producers at the moment belong to *Pleurosigma* and *Rhizosolenia* (Belt et al. 2000; Rowland et al. 2001)]. The similarities between the spatial distributions of biogenic silica and HBI III support an open-water diatom production signature for this proxy. Indeed, the distribution of HBI III reflects the phytoplankton bloom occurring in end-July/August in the fjord, as seen by the fluorescence values (Figs. 3, 4) and corresponds to the overall trend in increasing diatom dominance and abundance in the middle and outer fjord areas, reported from phytoplankton samples (Krawczyk et al. 2015). Interestingly, the highest HBI III value, measured in sediments from YST9, corresponds to the site where the highest diatom abundances have been found in the water column (Krawczyk et al. 2015).

It has been suggested that HBI III-producing diatoms bloom in higher abundance in open-water areas near the sea ice edge (Belt et al. 2015; Smik et al. 2016) due to enhanced mixing and nutrient availability at the surface. Although turbidity and dissolved silica availability are certainly important factors shaping the distribution of HBI III in zone B, the proximity of the ice edge in mid-July in this area is noteworthy, as this is a stable feature of the annual sea ice evolution in the fjord (Fig. 2). Similarly, the ice-edge position may also be reflected by the relatively high HBI III values at stations YST1 and YST2 (Fig. 6).

## Summary and conclusions

Primary production proxies (organic carbon and biogenic silica), and sea ice-related biomarkers (IP_25_ and HBI III) were analysed from 13 core-top samples along a transect from the outer Young Sound to the inner Tyrolfjorden. These proxies showed an overall good reflection of present-day conditions in the fjord, and a clear integration of the multiple factors (land–ocean–ice interactions) affecting this High Arctic ecosystem.

Organic carbon contents were <1.5% and followed a clear gradient, with the highest values closer to the fjord entrance (except for the shallower sites, where organic matter oxidation is more intense). Biogenic silica—a proxy for siliceous, mainly diatom production—was generally higher at the outer fjord stations and sites in the middle part of the ford, where there is a combination of high input from the glacial rivers (source of dissolved silica), lower turbidity than in the innermost fjord region, and some degree of exchange with more saline and nutrient-rich waters from the outer fjord/Greenland Sea. The biomarker HBI III followed a pattern roughly similar to that of biogenic silica and appears to reflect diatom production during the ice-free period, notably the annual bloom that starts outside the fjord mouth and penetrates into the middle part of the fjord. Nonetheless, higher concentrations of this biomarker were found near the mid-July ice edge. The sea ice proxy IP_25_ was present at all sites in the fjord, reflecting the seasonal character of the ice cover. However, IP_25_ concentrations were rather low (<40 ng g^−1^) with the highest concentration in the outer part of the fjord. This indicates that the fjord environment is somewhat unfavourable for the growth of sea ice diatoms producing IP_25_. This is possibly a consequence of poor light availability due to snow cover and low salinity levels resulting from the high runoff and meltwater input. We thus recommend caution when attempting to reconstruct past sea ice variability in fjord systems using IP_25_ records, as our results clearly indicate that these cannot simply be extrapolated to open ocean conditions. It is expected that future warming and enhanced melt in this region will lead to changes in sea ice cover, exchange with the ocean, and an increased runoff from land—with an unpredictable impact on primary productivity in the fjord, also for sea ice-related biota. This set of proxies, applied to sediment records covering past warm climate periods (such as the Medieval Warm Period and the Holocene Climate Optimum) have the potential to constrain future scenarios for the Young Sound region in a warming climate.

## Footnotes

1
www.asp-net.org.
